# The importance of thiamine (vitamin B1) in humans

**DOI:** 10.1042/BSR20230374

**Published:** 2023-10-10

**Authors:** Małgorzata Mrowicka, Jerzy Mrowicki, Grzegorz Dragan, Ireneusz Majsterek

**Affiliations:** Małgorzata Mrowicka, Jerzy Mrowicki, Grzegorz Dragan, Ireneusz Majsterek, Department of Clinical Chemistry and Biochemistry, Medical University of Lodz, Mazowiecka 5, 92-215 Lodz, Poland

**Keywords:** antioxidant properties, biological functions, deficiency in thiamine: causes and effects, neuropathy, thiamine, thiamine esters

## Abstract

Thiamine (thiamin, B1) is a vitamin necessary for proper cell function. It exists in a free form as a thiamine, or as a mono-, di- or triphosphate. Thiamine plays a special role in the body as a coenzyme necessary for the metabolism of carbohydrates, fats and proteins. In addition, it participates in the cellular respiration and oxidation of fatty acids: in malnourished people, high doses of glucose result in acute thiamine deficiency. It also participates in energy production in the mitochondria and protein synthesis. In addition, it is also needed to ensure the proper functioning of the central and peripheral nervous system, where it is involved in neurotransmitter synthesis. Its deficiency leads to mitochondrial dysfunction, lactate and pyruvate accumulation, and consequently to focal thalamic degeneration, manifested as Wernicke’s encephalopathy or Wernicke–Korsakoff syndrome. It can also lead to severe or even fatal neurologic and cardiovascular complications, including heart failure, neuropathy leading to ataxia and paralysis, confusion, or delirium. The most common risk factor for thiamine deficiency is alcohol abuse.

This paper presents current knowledge of the biological functions of thiamine, its antioxidant properties, and the effects of its deficiency in the body.

## Introduction

Vitamin B1, thiamine, is a vitamin with a multidirectional action on the human body and has long been of great interest to doctors, nutritionists and scientists. Thanks to its high biological activity and its role as an enzyme cofactor, thiamine has both direct and indirect effects on cell metabolism. Its deficiency in the human diet results in disturbances in many important biochemical and metabolic processes, such as an impaired glucose metabolism, disrupted bioenergetic processes, mitochondrial dysfunction, lactic acidosis (the consequence of pyruvate dehydrogenase [PDH] dysfunction in mitochondria), insufficient DNA synthesis due to low transketolase activity and ribose-5-phosphate synthesis in the pentose phosphate pathway and impaired neurotransmitter synthesis. Clinically, deficiency result in various pathologies, most notably, beriberi, a cardiomyopathy with edema and lactic acidosis, and Wernicke–Korsakoff syndrome or Wernicke’s encephalopathy. Thiamine deficiency is also implicated in several neurodegenerative diseases, such as Alzheimer’s disease, Parkinson’s disease, and Huntington’s disease [1].

## Thiamine availability

Only plants, microorganisms and some fungi are able to biosynthesize thiamine. For humans and animals, thiamine is an exogenous substance and it must be supplied to the body with food. Intestinal bacteria can also be a source of thiamine. Although some bacterial taxa, such as the Bacteroidetes and Actinobacteria, can synthesize vitamin B1 [2,3], these amounts are insufficient for a human being and hence should be acquired from a varied diet.

### Evolutionary factors behind animals losing the ability to synthesize thiamine

Many animals have lost the ability to synthesize thiamine, and therefore require it as part of their diet. One reason for this is the availability of thiamine in their natural diets. Thiamine is found in both plants and animals, and is readily available in the diets of many animals, making it unnecessary for them to synthesize it themselves. In fact, some animals, such as sheep and goats, rely on rumen microbes to produce thiamine for them. Therefore, the ability to synthesize thiamine has been lost in many animals due to the abundance of thiamine in their natural diets [4].

Another reason why animals have lost the ability to synthesize thiamine is due to reduced energy costs and metabolic requirements. The synthesis of thiamine requires a significant amount of energy and resources, which can be costly for an animal’s metabolism. By relying on external sources of thiamine, animals can conserve energy and resources, which can be better utilized for other metabolic processes [5]. Therefore, the loss of the ability to synthesize thiamine can be seen as an evolutionary adaptation to reduce energy costs and metabolic requirements.

Lastly, genetic adaptations have played a role in animals losing the ability to synthesize thiamine. As animals have adapted to rely on external sources of thiamine, their genes have changed to reflect this dependence [6]. In some cases, the loss of thiamine synthesis has been the result of mutations in genes that are responsible for the biosynthesis of thiamine. Additionally, recent studies have shown that thiamine-dependent enzymes play an important role in cancer cell metabolism, suggesting that genetic adaptations to depend on external sources of thiamine may have implications for disease susceptibility [7]. Overall, the loss of the ability to synthesize thiamine in animals is a complex process that involves a combination of factors, including the availability of thiamine in natural diets, reduced energy costs and metabolic requirements, and genetic adaptations.

## The structure and occurrence of thiamine

Thiamine, also known as thiamine and aneurine, was the first B vitamin to have been identified, thus its designation B_1_. It’s discovery was followed by riboflavin (B_2_), niacin (PP) (B_3_), choline (B_4_), pantothenic acid (B_5_), pyridoxine (B_6_), biotin (B_7_), inositol (B_8_), folic acid (B_9_), p-aminobenzoic acid (PABA) (B_10_), cobalamin (B_12_), orotic acid (B_13_), pangamic acid (B_15_), and laetrile (B_17_). Like all water-soluble vitamins, it is rapidly expelled through the urinary system, does not accumulate in the body, and has no toxic effects [8].

**Figure 1:**
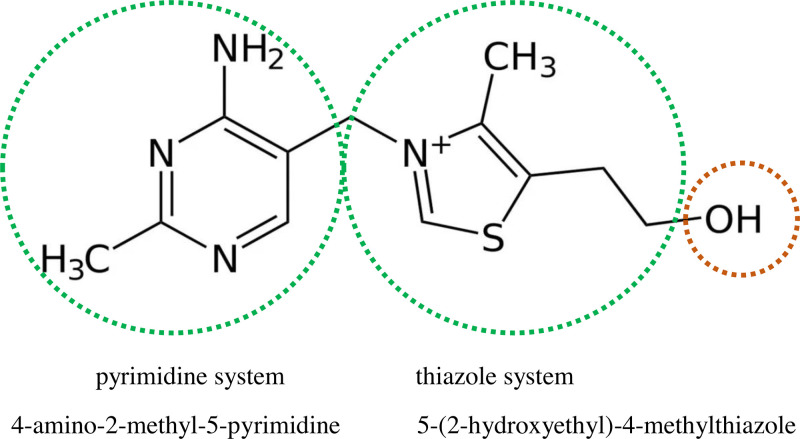
The structural formula of the thiamine molecule The underlined hydroxyl group (-OH) is the point of attachment of a phosphate group, which enables the formation of biologically active derivatives.

The thiamine molecule is composed of two heterocyclic rings, forming a pyrimidine and thiazole system connected to each other by a short methylene bridge. Due to the unbalanced positive charge located on the nitrogen atom in the thiazole ring, the entire molecule has a positive charge and forms thiazolonium salts (Figure 1). The chemical name for this water soluble vitamin is 3-[(4-amino-2-methyl-5-pyrimidinyl)methyl]-5-(2-hydroxyethyl)-4-methylthiazolium. While thiamine is normally found in its free form in plants, it tends to occur form as diphosphotiamine in animals (pyrophosphate, cocarboxylase) [9,10].

Thiamine exists in cationic form (T^+^) at physiological pH (Figure 1). In the intestinal lumen, before absorption, dietary thiamine is converted to free thiamine by intestinal phosphatases.

**Figure 2:**
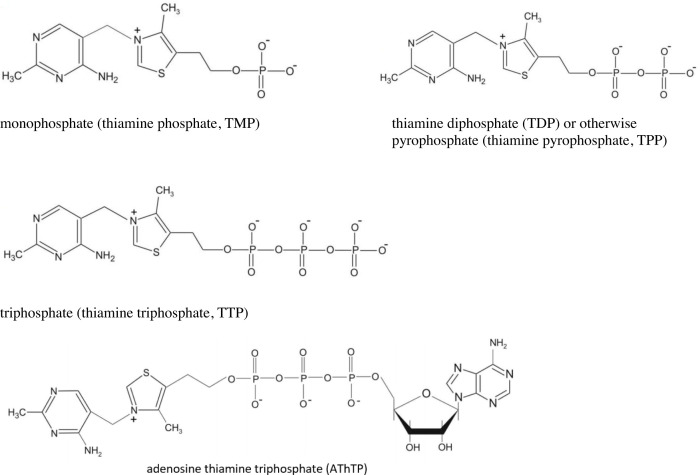
Structural formulas of phosphorylated thiamine derivatives

For transfer across the basal membrane of the enterocyte, T^+^ is converted into thiamine pyrophosphate (TPP) by thiamine pyrophosphokinase-1 (TPK1) and dephosphorylation of TPP to thiamine monophosphate (TMP) and thiamine by prostatic acid phosphatase (ACPP) [11]. In addition, in the human body, thiamine also occurs in the form of pyro- and triphosphate esters: thiamine diphosphate (TDP), pyrophosphate (TPP) and triphosphate (TTP) (Figure 2), as well as a recently discovered adenyl derivative of thiamine triphosphate, called adenosine triphosphotiamine (AThTP) [10,12].

Thiamine monophosphate (TMP) is usually present in cells in low concentrations and is considered to be biologically inactive. The highest concentrations of TMP in relation to total thiamine and its phosphorylated derivatives are found in nervous tissue animals and humans, 6–7%. It is the first two-ring product of the thiamine cation and plays an important role in maintaining the correct amount of thiamine in the cell, thus determining their direct availability. In cells, it is quickly converted to free thiamine by phosphatases. The physiological role of thiamine monophosphate is as yet unknown. It is considered an intermediate in the transformation pathways of phosphorylated thiamine derivatives [10].

**Figure 3:**
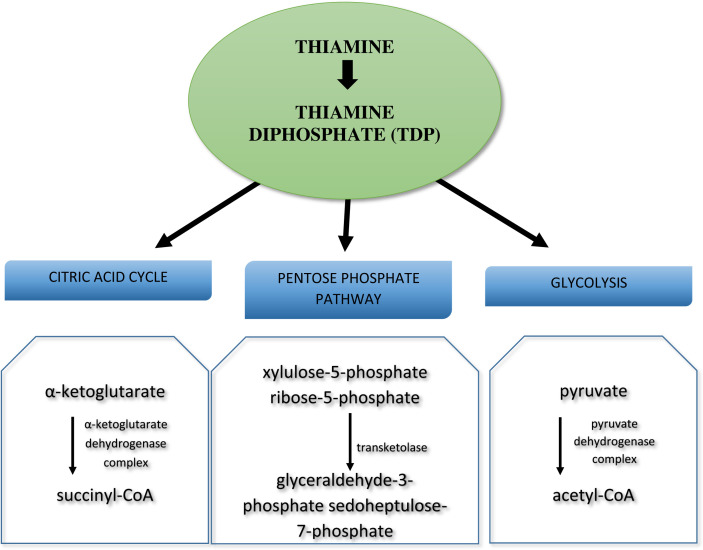
The major biochemical pathways involving thiamine

By far the best known derivative of thiamine is TDP (thiamine diphosphate), also known as TPP (thiamine pyrophosphate), which plays an important role as a cofactor for many key cellular enzymes. TDP is derived from the phosphorylation of free thiamine by thiamine pyrophosphokinase-1 (TPK1), dephosphorylation of triphosphate thiamine, adenosine triphosphotiamine breakdown combined with dephosphorylation or coenzyme release from proteolysable enzymes. TDP is the biologically active form of thiamine and serves as a cofactor for four major enzyme systems: (1) pyruvate dehydrogenase (PDH; EC 1.2.4.1) complex, the enzyme complex is responsible for central step in energy production, catalysing the reaction that links glycolysis with the tricarboxylic acid cycle (TCA); (2) α-ketoglutarate dehydrogenase complex (KGDHC; EC 1.2.4.2), a multicomponent enzyme complex, which binds specifically to complex I of the mitochondrial respiratory chain and is associated with the TCA enzyme supercomplex; the KGDHC is composed of multiple copies of three enzymes: α-ketoglutarate dehydrogenase (E1k component, EC 1.2.4.2), dihydrolipoamide succinyltransferase (E2k component, EC 2.3.1.12), and dihydrolipoamide dehydrogenase (E3 component, EC 1.6.4.3).; (3) transketolase (TKT; EC 2.2.1.1) plays an important role in the metabolism of pentose by producing ribose for nucleic acid synthesis and NADPH for the synthesis of fatty acids and steroids; (4) branched chain α-ketoacid dehydrogenase (BCKDH; EC 1.2.4.4) complex; the rate-limiting enzyme of branched chain amino acid catabolism [13]. The major biochemical pathways involving thiamine is summarized in Figure 3.

Interestingly, in addition to its cofactor function, thiamine derivatives also play non-coenzymatic roles. In microorganisms and plants TDP can be involved in the regulation of protein expression, being a major component of specific systems called riboswitch. These are mRNA fragments that contain specific binding sites for selected cellular metabolites that, when attached, can act as transcriptional terminators or translation inhibitors. TDP binding riboswitches have been identified within the mRNA of the following genes involved in thiamine biosynthesis: *thiM* in *Bacillus subtilis*, *thiM* and *thiC* in *Escherichia coli*, and *THIC* in *Arabidopsis thaliana, Oryza sativa*, and *Poa secunda* plants [10,14,15].

Literature data indicate that more than 20 different thiamine pyrophosphate-dependent enzymes are known, most of which are found in prokaryotes: transketolase [16], phosphonopyruvate decarboxylase [17], pyruvate dehydrogenase complex [18], branched-chain amino acid enzyme [19], phosphoketolase [20], benzoylformate decarboxylase [21], pyruvate decarboxylase [22], 2-oxoglutarate dehydrogenase complex [23], sulfopyruvate decarboxylase [24], pyruvate ferredoxinoxidoreductase [25], phenylpyruvate decarboxylase [26], 2-hydroxyphytanoyl-CoA lyase [27], acetohydroxyacid synthases [28], glyoxylate carboligase [29], and others.

TDP in the cytosol is tightly bound by transketolase and other thiamine-dependent enzymes [30]. In all its reactions, TDP deprotonates the carbon at the C-2 position of the thiazole ring to form an ylid 2-carbanion. Decarboxylation is initiated by the formation of a C-2 carbanion of TDP, which interacts with the C-2 of α-ketoacids to form a nucleophilic adduct, followed by CO_2_ release and formation of the C-2a-carbanion/enamine. Subsequent protonation leads to the formation of an *active aldehyde* intermediate whose metabolism depends on the specific enzyme involved. Finally, all α-ketoacid decarboxylases facilitate decarboxylation reactions with the subsequent release of an aldehyde molecule; this forms part of a range of metabolic pathways. Moreover, in the presence of a further aldehyde or 2-ketoacid molecule acting as an acceptor, the C-2a-carbanion/enamine yields 2-hydroxyketones or hydroxyketoacids [31].

Thiamine diphosphate-dependent reactions are integral to bioenergetics in a range of microorganisms, including prokaryotes and yeast, and play a key role in various processes such as alcoholic fermentation, oxidative phosphorylation, and substrate level phosphorylation. Additionally, these reactions are also involved in many anabolic reactions such as photosynthesis, fatty acid, isoprenoid, and nucleotide biosynthesis [5].

In contrast, both thiamine triphosphate (TTP) and adenosine triphosphotiamine (AThTP) appear to be involved in non-co-enzymatic reactions. TTP plays influences membrane conductance through activation of the voltage-gated Cl^−^ anion channels with low specificity, and functions as a phosphate group donor in phosphorylation reactions in energy metabolism.

Thiamine triphosphate may be involved in the phosphorylation of associated proteins with synaptic signaling, including the protein rapsin 43K, which is associated with the nicotinic acetylcholine receptors (nAChR) in the postsynaptic membrane [32]. In addition, TTP has been found to phosphorylate other proteins, which could confirm that this molecule plays a regulatory role and may be involved in signaling pathways [11]. TTP and AThTP molecules are primarily responsible for ensuring signal functions under stress conditions. They have been found to accumulate in response to carbon and/or nitrogen deficiency in bacteria and in human cells [33–35].

## Thiamine homeostasis

Thiamine is absorbed to the greatest extent by the intestinal walls, where T^+^ it is transferred to the blood, where its target places are erythrocytes, plasma, leukocytes and platelets [36,37].

Subsequently, thiamine is taken up by cells within various tissues including the liver and heart from the blood; however, in the case of neuronal tissue, thiamine is transported from the blood into the cerebrospinal fluid via the blood–brain barrier (BBB). Once within the cell, further transport occurs through mitochondrial and nuclear membranes. Proteins with affinity for organic ions are involved in thiamine transport, including pyrophosphokinase, solute carrier proteins (SLC), alkaline phosphatase transport system (ALP) and organic cation transporter proteins (OCTs) [38].

Proteins from the SLC19 family, a subgroup of proteins from the SLC (solute carrier protein) superfamily, also participate in thiamine and folate transport. Three proteins from the SLC19A membrane transporter family are responsible for the transport of thiamine to animal tissue cells.

**Figure 4:**
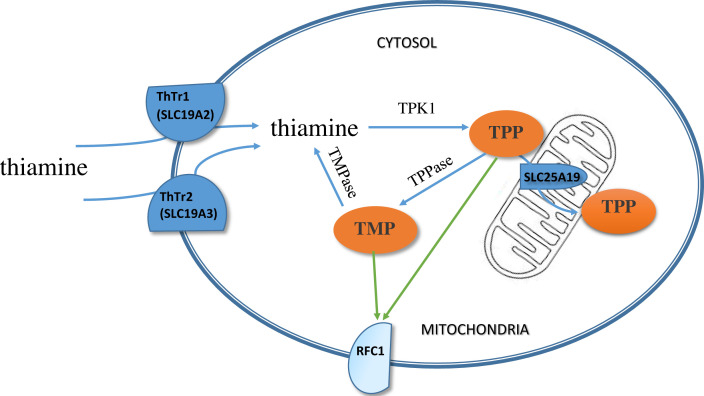
Schematic diagram of thiamine membrane transport ThTr1, SLC19A2: thiamine transporter-1; ThTr2, SLC19A3: thiamine transporter-2; RFC1: reduced folate carrier; TPP: thiamine pyrophosphate; TMP: thiamine monophosphate; TPK1: thiamine pyrophosphokinase; TMPase: thiamine monophosphate phosphatase; TPPase: thiamine pyrophosphate phosphatase

The reduced folate carrier (RFC, SLC19A1), thiamine transporter-1 (ThTr1, SLC19A2) and thiamine transporter-2 (ThTr2, SLC19A3) transmembrane proteins differ. The RFC carries thiamine monophosphate, pyrophosphate and triphosphate across cell membranes from the inside of the cell, contributing to the maintenance of homeostasis of phosphorylated vitamin B1 derivatives [39,40]. The reduced folate carrier (RFC) is folate-specific and does not transport T^+^, but functions as an anion exchanger (Figure 4).

ThTr1 and ThTr2 transport T^+^ into the cell across its membranes but do not bind folates. Thiamine absorption is mainly mediated in the upper small intestine by two highly specific transporters, ThTr1 andThTr2, the respective products of the *SLC19A2* and *SLC19A3* genes [11,41].

Another physiologically important family member is the thiamine pyrophosphate transporter TPC (SLC25A19) which carries the cofactor needed for pyruvate dehydrogenase (PDH) and α-ketoglutarate dehydrogenase (α-KGDH) across the mitochondrial membrane [42].

In addition, human extraneuronal monoamine transporters (hEMT) transport the amine forms of nutrients and neurotransmitters, including thiamine, to the neurons [43]. Another set of thiamine transporters are alkaline/acid phosphatases (ALPs), which are membrane-bound metalloenzymes present in the intestine, liver, kidney, heart, bone and placenta. ALPs transport thiamine to the cytosol by dephosphorylating them to T^+^ [44].

Once inside the liver and brain cells, thiamine is phosphorylated by a specific enzyme, thiamine pyrophosphokinase-1 (TPK1), to its active form, thiamine diphosphate (TDP), a cofactor for several enzymes involved in energy metabolism. Phosphorylation of thiamine by TPK1 has been shown to be a significant driving force for thiamine uptake. Human TPK1 exists as a homodimer and is present in all types of human cells, with high levels in the kidney, small intestine, and testis [45]. TDP can either be phosphorylated further to thiamine triphosphate (TTP) by the enzyme TDP phosphoryl transferase, or dephosphorylated to thiamine monophosphate (TMP) by the enzyme thiamine diphosphatase (TDPase). In addition, dephosphorylation of intracellular TPP to TMP can be subsequently recycled back to free thiamine via thiamine monophosphatase [11].

Thiamine is well soluble in water and holds relatively low affinity for serum proteins, allowing efficient filtration in the glomeruli and excretion in the urine. The filtration rate of thiamine in the kidneys is proportional to its concentration in the blood: greater reabsorption is noted in the kidneys at low vitamin B concentrations in the blood. Excess free thiamine and TMP are excreted in urine [46].

## Thiamine: bioavailability and requirement

Dietary thiamine requirements change with age, sex, and physiological condition (sedentary or active lifestyle). The daily requirement of an adult human is in the range of 1.1–1.5 mg/day. The standards for thiamine, established at the level of EAR (Estimated Average Requirement) and RDA (Recommended Dietary Allowances) are summarized in Table 1. Thiamine is found in plant-derived foods (mainly in the form of thiamine monophosphate), including enriched bread and whole grain cereals, peas, beans, nuts and brown rice, and in animal products (mainly in the form of thiamine pyrophosphate) such as meats, especially pork loin, pork shoulder and beef. Thiamine is sensitive to high temperature, especially at pH values above 5. Its losses during technological and culinary processing of food range from 20 to 70% [47]. Vitamin retention depends on the pH of the environment in which processing takes place. Thiamine, folates and vitamin C are sensitive to neutral environments, while retinol, β-carotene and folates are sensitive to the acid environments, and vitamin D, thiamine, riboflavin and vitamin C to alkaline ones [48].

**Table 1:** Age- and sex-dependent recommended EAR and RDA of thiamine (based on Jarosz et al., 2020) [47]

Group sex, age (years)	mg thiamine/person/day	
	EAR	RDA
**Infants and children**		
0–0.5	0.2	
0.5–1	0.3	
1–3	0.4	0.5
4–6	0.5	0.6
7–9	0.7	0.9
**Adolescents and adults**		
10–12	0.8–0.9	1.0
13–15	0.9–1.0	1.2
16–18	0.9–1.0	1.2
**Men**		
19–75	1.1	1.3
>75	1.1	1.3
**Women**		
19–75	0.9	1.1
>75	0.9	1.1
**Pregnant women**		
<19	1.2	1.4
≥19	1.2	1.4
**Breastfeeding women**		
<19	1.3	1.5
≥19	1.3	1.5

EAR, Estimated Average Requirement; RDA, Recommended Dietary Allowances.

Ingested thiamine from food and dietary supplements is absorbed by the small intestine through active transport at nutritional doses and by passive diffusion at high concentrations. Thiamine absorption in the jejunum involves both passive diffusion and active transport, with thiamine being converted into thiamine pyrophosphate (TPP). At high concentrations, thiamine can pass through the intestinal membranes by spontaneous diffusion. Once absorbed, thiamine is transported to various tissues, including erythrocytes, through passive diffusion and active transport mechanisms [49]. Human enterocytes have the ability to absorb thiamine and its phosphate salt. The intestinal enzyme phosphatase hydrolyzes thiamine into a free form which is then absorbed by the small intestine. The total amount of vitamin B_1_ in the body is 30 mg, 40% of which is in the muscles. The phosphorylated form of thiamine gets stored in the brain, heart, liver and kidneys. As thiamine has a short half-life of only one to 12 h, it is important to consume it regularly in the diet [50]. If not, thiamine storage is depleted within 2–3 weeks. Accelerated thiamine metabolism is observed in hypermetabolic states, e.g., during withdrawal from alcohol, convulsions, infections, diabetes, or a rapidly developing neoplastic disease, especially of the gastrointestinal tract and the hematopoietic system [51–53].

## Influence of thiamine on the nervous system

Thiamine plays an enzymatic and non-enzymatic role in the nervous system. The symptoms of thiamine deficiency are mainly associated with decreased activity of enzymes whose cofactor is thiamine diphosphate. These symptoms are first observed in the nervous system. This is explained by the toxic effect of lactate accumulating due to the reduced activity of the pyruvate dehydrogenase complex, overproduction of free radicals and oxidative stress, and changes in the microglia at the onset of neurodegeneration [54–58]. Thiamine deficiency reduces the level of synthesis of nucleic acids, fatty acids and steroids, which in turn causes demyelination of nerve fibers.

Thiamine also plays a nonenzymatic role as an active component of the axoplasmic, mitochondrial and synaptosomal membranes. Thiamine is involved in the processes of cellular differentiation, synapse formation, axonal growth, and myelinogenesis [9].

### Thiamine and neurotransmitters

Thiamine is involved in glucose metabolism, maintains the functions of the nerve membrane and supports the synthesis of myelin and several neurotransmitters, e.g., acetylcholine, serotonin, and amino acids (aspartate and glutamate) [9]. Thiamine has been shown to inhibit the activity of acetylcholine esterase (AChE): an enzyme that breaks down one of the most important neurotransmitters, acetylcholine, into choline and acetic acid. Supplementation with thiamine increases AChE activity. Therefore, thiamine is an important in enzymatic processes involved in brain development, brain function, maintenance, and interneuronal communication. The form of cationic thiamine (T**^+^**) influences the action potential of nerves, membrane conductivity and transmission of neuronal signals, and stimulates the activity of the spinal cord and cerebellum [59–61]. Thiamine is involved in serotonin uptake, and indirectly in the activity of the cerebellum, hippocampus and hypothalamus [62,63]. Vitamin B1 facilitates the uptake of GABA (γ-butyric acid) neurotransmitter, and its deficiency results in cell damage, affecting cerebellum activity [64]. Moreover, thiamine is involved in maintaining the proper structure of the myelin sheaths, and therefore contributes to the speed of nerve conduction [65].

### Thiamine and mood

The vitamin B1 prevents depression and has a positive effect on well-being. A direct correlation was first noted between thiamine deficiency and symptoms of depression in a study of 74 patients with a history of malnutrition [66]. A cross-sectional study on >1500 Chinese people aged 50–70 years showed that subjects with lower concentrations of thiamine displayed more severe symptoms of depression [67]. In addition, a study by Ghaleiha et al. [68] showed that depression symptoms significantly improved in patients with major depressive disorder after six weeks of thiamine supplementation compared with placebo.

## Thiamine deficiency

The total blood concentration of thiamine and its derivatives is 1 μM in animals, but only 0.1 μM in humans [11]. Distribution of thiamine derivatives in human fluids is provided in Table 2.

**Table 2:** Distribution of thiamine derivatives in human whole blood and plasma in nmol/L ± standard deviation (SD) based on Gangolf et al. [1]

[nmol/L] ± SD	Samples (*n*)	
	Whole blood (7)	Plasma (3)
Thiamine	4 ± 3	11 ± 3
TMP	10 ± 4	5 ± 2
TDP	138 ± 33	n.d.
TTP	13 ± 4	n.d.

n.d., not detectable; TDP, thiamine diphosphate; TMP, thiamine monophosphate; TTP, thiamine triphosphate.

Insufficient intake can lead to thiamine deficiency within 18 days [69]. Hence, humans are highly susceptible to thiamine deficiency. This may result from (I) poor intake, e.g., chronic alcoholism, inappropriate diet, gastric bypass surgery; (II) poor absorption, e.g., gastric bypass surgery, vomiting, neoplastic hyperplasia, malnutrition, malabsorbtion syndrome; (III) increased loss, e.g. diarrhea and vomiting, hemodialysis, diuretic drug use, systemic illness, infection/sepsis and (IV) poor utilization, e.g., pregnancy, lactation, decreased enzyme activity, and hyperthyroidism [70].

Metabolic disturbances in thiamine deficiency lead to metabolic acidosis, and laboratory evaluation will often reveal an elevated lactate concentration. These symptoms mainly include muscle cramps and pain, problems with memory and concentration, recurrent fatigue, depression, swelling of the limbs, decreased libido, abnormal digestive system, excessive weight loss and increased heart rate, and nystagmus. When thiamine is excluded from the diet for a long time, neurological disorders may develop. Beriberi disease may also develop, resulting in skeletal muscle atrophy, weakening of the contractile strength of the heart muscle, reduced blood pressure and paralysis of the nervous system. Diagnosing beriberi is difficult because it is based exclusively on the observation of clinical symptoms. Vitamin B1 deficiency can also result in Korsakoff’s syndrome and apathy.

There are two clinical types of avitaminosis: wet and dry. The former is accompanied by extensive edema, resulting in an abnormal cardiovascular system, circulatory failure and heart attack, while the latter is associated with abnormal functioning of the nervous system and the development of polyneuropathy and Wernicki’s encephalopathy. The most characteristic symptoms are eye movement disorders, impaired consciousness and ataxia. In the acute form of beriberi disease, called Shoshin syndrome, cardiovascular failure, metabolic acidosis, and pulmonary edema may occur. Thiamine deficiencies are also associated with aging-related disorders such as Parkinson’s, Alzheimer’s, kidney disease, cancer, mental disorders, and other diseases of the cardiovascular and nervous systems [71,72]. Supplementation with high doses of thiamine reduces the intensity of Alzheimer’s [73] and Parkinson’s [74] symptoms. In patients with Alzheimer’s disease (AD), thiamine levels and the activity of thiamine-dependent enzymes are reduced in both brain and peripheral tissues. However, there is evidence linking abnormalities in thiamine availability and metabolism to the pathophysiology of AD. Thiamine has been suggested to have a beneficial effect in the treatment of AD, but further investigation is needed to determine its efficacy [75].

Studies investigating the efficacy of thiamine in the treatment of AD have shown promising results. Oral thiamine trials have been shown to improve cognitive function in patients with AD [76]. Additionally, a study by Gibson et al. in the 1990s found that high-dose thiamine was effective in treating AD [77]. Furthermore, a recent study by Sang et al. found that thiamine metabolite levels in blood samples were correlated with brain glucose hypometabolism in AD [78]. The optimal dosing and administration of thiamine for the treatment of AD is still being investigated. A study by Dingwall et al. found that intravenous administration of 100 mg thiamine hydrochloride in a 100 ml bag of normal saline was effective in treating patients with Wernicke–Korsakoff syndrome, a condition caused by thiamine deficiency [79].

Thiamine also supports the action of non-steroidal anti-inflammatory drugs in back pain syndromes or in pain after limb fracture. It has been shown that thiamine can relieve pain by inhibiting the activity of thalamic and spinal neurons, affecting the effectiveness of the neurotransmitters noradrenaline and 5-hydroxytryptamine [80–82].

Secondary thiamine deficiency is caused by increased requirement, as in pregnancy, lactation, fever and hyperthyroidism. However, it is also associated with impaired absorption, as in prolonged diarrhea, and impaired utilization, as in severe liver disease. In addition, gastrointestinal dysfunction and increased urinary loss, septic shock, viral infections, hemodialysis, and alcohol abuse can further reduce thiamine concentrations. Since alcohol is the most common risk factor in adults, it is possible that in newborns develop thiamine deficiency as a result of fetal alcohol syndrome [83–94].

Moreover, the main causes of thiamine deficiency in young children include poor dietary intake, malabsorption, and underlying medical conditions such as anorexia, morbid obesity, and bariatric surgery. In young children, thiamine deficiency can lead to a range of neurological signs and symptoms. These can include cognitive problems, decreased alertness, difficulty breathing, heart problems, muscle weakness, and problems with memory and vision. In severe cases, thiamine deficiency can even lead to coma and death. The classic presentation of thiamine deficiency is known as beriberi, which is characterized by nerve involvement and can lead to symptoms such as ataxia, oculomotor abnormalities, and aphonia. Early diagnosis and treatment of thiamine deficiency can help prevent serious health consequences and improve outcomes for young children [94–96].

It is also important to understand the effect of thiaminases on thiamine deficiency in food is crucial in preventing thiamine deficiencies and maintaining adequate levels of this essential nutrient in the human diet. Thiaminases are enzymes that break down thiamine in food products such as tea, coffee, raw fish, and shellfish, and can cause depletion of thiamine in these foods [97]. Thiaminases work by breaking down the thiamine molecule into inactive components. The mechanism of thiamine depletion in food involves the activity of two types of thiaminases, thiaminase I and thiaminase II [98]. Thiaminase I is found in some bacteria and plants and can degrade thiamine in food products, while thiaminase II is found in some fish and can cleave the thiamine molecule into inactive components. Understanding these factors can help prevent thiamine depletion in food and ensure adequate levels of this essential nutrient in the diet [99,100].

Moreover, oxidative stress and systemic inflammation can rapidly decrease thiamine reserves [101].

## Role of oxidative stress in thiamine deficiency (TD)

In all tissues, thiamine acts as an important cofactor for key enzymes in the glucose, amino acid, and lipid metabolisms. The brain, consuming approximately 20% of the oxygen needed for survival, is particularly vulnerable to attack by free radicals. Almost 5% of the oxygen that is used in the respiratory chain, mitochondria and peroxisomes is converted into its reactive oxygen forms (ROS) such as superoxides, hydrogen peroxides, singlet oxygen, and hydroxyl ions. Oxidative stress is also generated by reactive nitrogen species (RNS), which includes nitrate, nitrite, nitric dioxide, nitric oxide, and peroxynitrite. Oxidative stress in the brain may occur in response to overproduction of both ROS and RNS, decreased activity of antioxidant enzymes such as superoxide dismutase (SOD), catalase (CAT), glutathione peroxidase (GPx), and/or decreased concentration of reducing agents [102,103].

Oxidative stress may reduce the levels of thiamine, thiamine phosphates and thiamine-dependent enzymes. The reduction in thiamine or thiamine phosphates could enhance the oxidative imbalance and lead to neurodegeneration. The proper functioning of neurons requires an enormous energy supply, provided by mitochondrial ATP synthesis. Moreover, the mitochondria also control intracellular Ca^2+^ homeostasis, which are essential for the regulation of neurotransmitter exocytosis, and in the regulation of gene expression. It also regulates thermogenesis, cell division and apoptosis. Mitochondria are also sources of ROS, particularly superoxide anions (O2^•−^) [104,105].

Additionally, TD decreases the activity of thiamine-dependent enzyme, particularly the α-KGDH complex, which leads to mitochondrial dysfunction, resulting in decreased activity of the tricarboxylic acid cycle in endothelial cells, astrocytes, and microglia. In the brain, most sensitive area to TD, excessive oxidative stress, and inflammation process is the sub-medial thalamus nucleus. Numerous markers of oxidative stress occur in this region, including hydroxynonenal, a product of peroxidation; inducible, endothelial and neuronal nitric oxide synthase (iNOS eNOS, nNOS respectively), hemeoxygenase-1 (HO-1), intercellular adhesion molecule 1 (ICAM-1), CD40L and CD40L glycoproteins, astrocytes and microglia activation, tumor necrosis factor α (TNFα), and IL-1β and IL-6 [106–109].

Increased extracellular glutamate levels and thus activation of the non-ionotropic NMDA receptor happens due to the overproduction of ROS and RNS. This results in the typical features of TD, such as excitotoxicity and disruption of the blood–brain barrier (BBB), which allows the passage of many vascular factors from the systemic circulation to the brain, leading to the activation of microglia and the induction of apoptosis [40,110]. Moreover, free radicals generate many changes in the membranes of nerve cells, such as those occurring due to lipid peroxidation (such as polyunsaturated fatty acids—membrane phospholipids), modification of membrane enzyme activity, oxidation of membrane protein thiol groups, disturbance of membrane transport, change of the antigenic character of membranes and deregulation of membrane potential [111].

Because the endoplasmic reticulum (ER) is the site of calcium ion storage, TD and oxidative stress can cause dysregulation intracellular calcium homeostasis and may induce ER stress [112]. As demonstrated by Liu et al. [39], TD can lead to ER stress. It is caused by the accumulation of misfolded or unfolded proteins in the ER lumen, resulting in overexpression of ER stress markers such as growth arrest and DNA damage-inducible protein 153 (CCAAT/enhancer binding protein homologous protein), glucose-regulated protein 78 (GRP78), and C/-EBP homologous protein (CHOP). It also leads to the phosphorylation of the eukaryotic initiation factor 2α (eIF2α) and the cleavage of caspase-12.

## The antioxidant protective role of thiamine

In addition to the metabolic and structural function, thiamine has also demonstrated antioxidant properties. Thiamine as scavenger of ROS removes hydroxyl (HO^•^) radical significantly higher than hydroperoxyl radical (HOO^•^) [113].

Lukienko et al. [114] investigated the antioxidant effects of thiamine in rat liver microsomes, and its interaction with reactive oxygen species. Their results indicate that thiamine is protective against a variety of toxic agents that promote oxidative stress. The authors explain that in the presence of oxidants a thiol form of thiamine is oxidized to thiamine disulfide, tricyclic form to thiochrome. The antioxidant effect of thiamine is probably related to two-phase reaction of opening of thiazole ring with formation of anion of thiol form of thiamine and unstable tricyclic form. In an another study, the hydroperoxide generation in linoleic acid peroxidation was significantly decreased by thiamine hydrochloride [115].

Gliszczyńska-Świgło [116] examined the antioxidant potential of thiamine, folic acid, three forms of vitamin B6 (pyridoxine, pyridoxal, and pyridoxamine) using trolox equivalent antioxidant capacity (TEAC) assay and ferric reducing antioxidant power (FRAP) assay. The highest antioxidant activity was found for thiamine.

Thiamine also demonstrated oxidative reactivity with hypochlorous acid (HOCl) [117].

Thiamine has a protective effect against the development of the diseases associated with Fenton-mediated oxidative damage. The experiment showed that vitamin B1 protects hepatocytes from iron-catalyzed oxidative stress by decreasing lipid peroxidation, mitochondrial and protein damage, and DNA oxidation [118].

However, Hu et al. [119] reported thiamine to have adverse effects on other forms of oxidative stress: thiamine stimulates microsomal lipid peroxidation induced by FeCl_3_ and ascorbate. The authors suggest that the radical-scavenging ability of thiamine does not appear to correlate with its effects on microsomal lipid peroxidation.

A recent report [120] found that thiamine reduces neutrophil extracellular trap (NET) formation in a dose-dependent manner. Furthermore, co-treatment with oxythiamine, a transketolase inhibitor, and thiamine inhibits intracellular ROS generation and might contribute to the potential regulation of ROS-dependent NETosis. NET is an antimicrobial mechanism involving NETs. It composed of decondensed nuclear DNA and associated antimicrobial web-like granules that bind pathogens. NETs allow neutrophils to kill extracellular pathogens while minimizing damage to the host cells. The release of NETs can be influenced by ROS generated by NADPH oxidase [121]. Zawrotniak et al. [122] showed that thiamine can inhibit UV-induced netosis.

Moreover, thiamine has strong antioxidant properties, thanks to which it has anti-inflammatory and anti-tumor properties by increasing the phagocytic activity of macrophages. As an example of the antioxidant properties of thiamine, we can mention the inhibition of NF-κB (nuclear factor κ-light-chain-enhancer of activated B cells) stimulation caused by oxidative stress and the protection of the surface sulfhydryl groups of neutrophils against changes caused by oxidation; moreover, it contributes to the formation of proinflammatory cytokines in macrophages. It also interacts with the p53 suppressor protein, which is one of the most important proteins guarding the cell cycle, controlling proliferation, apoptosis and cell death. [123–125].

Moreover, thiamine is a potent inhibitor of advanced glycation end-product (AGE) formation by the non-enzymatic glycation of proteins by glucose [126]. These molecules contribute to both functional and structural modifications of many proteins, cellular dysfunctions and apoptosis, which leads to damage to tissues and organs. It can occur because of AGEs engagement in the production of reactive oxygen and nitrogen species, and also accompany oxidative stress and inflammation. Ultimately AGEs contribute to the pathological symptoms of diabetes mellitus, renal disease, cardiovascular disease, aging, and neurodegenerative diseases [127,128]. Thiamine has a protective effect against the development of the atherosclerotic plaque because it boosts endothelial functions and retards atherosclerosis progression [129].

An important report is the study on the immunological role of thiamine in T cells development in thymus, especially the development of double-negative (DN) cells into double-positive (DP) or γδ thymocytes. It has been shown that thiamine modulates the metabolism of branched-chain α-keto acids (BCKAs) in thymic stromal cells. The authors demonstrated that thiamine mediated interaction between stromal and immune cells for the appropriate development of thymocytes [130].

Moreover, thiamine is a potent anti-inflammatory modulator with antioxidant and anti-tumor properties that increases the phagocytic activity of macrophages [131,132].

Thiamine does not allow the expression of the Bcl-2 family of pro-apoptotic proteins, caspase-3 activation, and poly-(ADP-ribose) polymerase (PARP) cleavage. It alters mitochondrial membrane potential, release of cytochrome *c*, apoptosis-inducing factor, and phosphorylation [133].

The latest clinical observations on the link between thiamine and SARS-19 infection are also very interesting. Thiamine, in addition to its many properties protecting the immune system, plays a significant role in eliminating the SARS-CoV-2 virus by activating humoral and cellular immunity [134]. Therefore, sufficient doses of thiamine help maintain a healthy immune response during SARS-CoV-2 infection. One of the symptoms of COVID-19 is a pulmonary edema similar to that seen at high altitudes. Carbonic anhydrase isoenzyme inhibitors (e.g. acetazolamide) are used to prevent pulmonary edema, altitude sickness, and increase oxygen levels. Thiamine also acts as an inhibitor of the isoenzyme of carbonic anhydrase. Therefore, high doses of thiamine administered to patients in the early stages of COVID-19 have the potential to reduce hypoxia and hospitalization [135–137]. Besides, Al Sulaiman et al. [138] showed that thiamine was associated with a lower incidence of thrombosis.

In the cells of the immune system, the induction of an immune response (inflammation) is associated with a switch of metabolic energy production from glucose, from oxidative phosphorylation to aerobic glycolysis. Thiamine has general anti-inflammatory properties by dephosphorylating pyruvate dehydrogenase, which intensifies the conversion of pyruvate into acetyl-CoA; in addition, thiamine inhibits the breakdown of pyruvate to lactate. The effect of thiamine on individual types of cells of the immune system is not fully known, the works dealing with this topic are summarized in the table below (Table 3).

**Table 3:** The effect of thiamine on cells of the immune system

A type of the immune system cells	Action of thiamine	References
**T lymphocytes**	Thiamine is required for the proper differentiation of T lymphocytes in the cells of the thymus	Hirata et al. [130]
**Macrophages**	Thiamine affects M1 (pro-inflammatory) macrophages to M2 (anti-inflammatory) type	Ehmedah et al. [139]
**Dendritic cells**	Inhibition inflammatory processes by inhibiting oxygen glycolysis with the participation of allithiamine	Choi et al. [140]
**Neutrophils**	Thiamine can reduce the formation of extracellular neutrophils (NET) depending on ROS through antioxidant effects	Riyapa et al. [120]
**Thrombocytes**	Thiamine is involved in hematopoietic processes, with large tiamine deficiencies associated with, e.g., mutations in the *SLC19A2* gene; this can lead to thrombocytopenia	Moulin et al. [141]

## Conclusions

**Figure 5:**
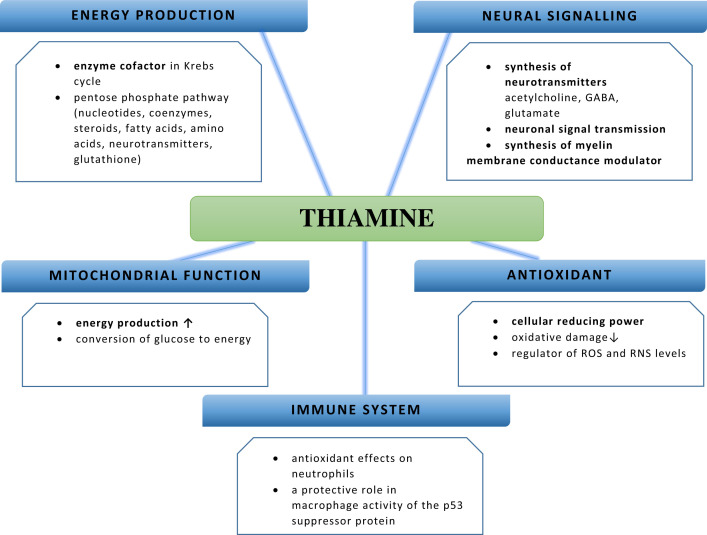
Physiological functions of thiamine

Our findings highlight the multidirectional biological activity of thiamine, as summarized in Figure 5. It plays an important coenzymatic and non-coenzymatic role in cell metabolism. It is a cofactor of many highly significant enzymatic reactions that control bioenergetic processes, amino acid metabolism and the transformation of other compounds organic, including pentoses necessary for the production of nucleotides. The phosphorylated thiamine derivatives also play a non-co-enzymatic role in controlling cell metabolism; this is made possible through allosteric regulation of related enzymes with cell bioenergetics, participation in transmission nerve signals at synapses and the potential participation in regulatory and signaling pathways related to receiving stimuli from the environment.

Since thiamine is an element of the pathways that produce reducing power in cells, its deficiency is tantamount to exposing cells to oxidative stress. This can lead to all kinds of oxidative damage to cells and their death, and thus, pathological symptoms and comorbidities. Therefore, it seems that thiamine homeostasis may reflect the oxidative state of cells. As such, thiamine is very important for the proper functioning of the nervous system due to its activating role on the excitability and metabolism of neurons and its antioxidant properties. Importantly, thiamine also specifically protects the brain.

Adequate dietary intake of thiamine ensures the proper functioning of tissues and organs. Although appropriate doses are important in all age groups, the elderly population, especially those affected by neurodegenerative diseases are particularly vulnerable to deficiency, which needs to be confirmed with appropriate diagnostic tests.

In any cases, deficiency may lead to serious health consequences, especially in the area of the nervous and cardiovascular systems. In many clinical cases, supplementation will prevent their deficiencies and obtain specific health benefits.

## Abbreviations

AThTP: adenosine triphosphotiamine
BCKA: branched-chain α-keto acid
DN: double-negative
DP: double-positive
FRAP: ferric reducing antioxidant power
HOCl: hypochlorous acid
NET: neutrophil extracellular trap
PABA: p-aminobenzoic acid
PARP: poly-(ADP-ribose) polymerase
PDH: pyruvate dehydrogenase
TEAC: trolox equivalent antioxidant capacity
TMP: thiamine monophosphate
TPK1: thiamine pyrophosphokinase-1
TPP: thiamine pyrophosphate
